# Psychometric properties of the Chinese version of Spiritual Index of Well-Being in elderly Taiwanese

**DOI:** 10.1186/s12877-016-0392-1

**Published:** 2017-01-04

**Authors:** Li-Fen Wu, Shu-Hui Yang, Malcolm Koo

**Affiliations:** 1 Department of Nursing, National Taichung University of Science and Technology, Taichung City, Taiwan; 2 Department of Nursing, Taichung Veterans General Hospital, Taichung City, Taiwan; 3 Department of Medical Research, Dalin Tzu Chi Hospital, Buddhist Tzu Chi Medical Foundation, 2 Minsheng Road, Dalin, Chiayi, 62247 Taiwan; 4 Dalla Lana School of Public Health, University of Toronto, Toronto, ON Canada

**Keywords:** Health surveys, Psychometrics, Questionnaires, Reliability and validity, Spirituality, Well-being

## Abstract

**Background:**

Spiritual well-being has become an increasingly important issue for the elderly people. The 12-item Spirituality Index of Well-Being (SIWB) is a well-validated instrument for assessing a patient’s current spiritual state. However, the psychometric properties of the SIWB in the Chinese elderly populations are not known. Therefore, this study translated the SIWB into Chinese and evaluated its psychometric properties.

**Methods:**

The English version of the SIWB was first translated into Chinese based on the Brislin’s translation model. The psychometric properties of the translated version of the SIWB (SIWB-C) was evaluated in 416 elderly Taiwanese recruited using a purposive sampling procedure from a medical center, a long-term care institution, and a community health center. Convergent validity was accessed using Pearson’s correlation coefficients of the SIWB-C, the EQ-5D-3 L health-related quality of life scale, and the Geriatric Depression Scale-5 (GDS-5). Exploratory factor analysis with Varimax rotation was performed to determine the construct validity. Confirmatory factor analysis was conducted for verification of the quality of the factor structures and demonstrating the convergent validity of the SIWB-C. An internal consistency test based on the Cronbach’s alpha coefficient and a stability test based on the Guttman split-half coefficient were also performed. Test-retest reliability was evaluated with intraclass correlation coefficient.

**Results:**

Exploratory factor analysis confirmed the original two-dimensional structure of the scale. Confirmatory factor analysis indicated a well-fitting model and a fine convergent validity of the SIWB-C. The Cronbach’s alpha coefficient and the Guttman split-half coefficient for the SIWB-C were 0.94 and 0.84, respectively. The correlations between the SIWB-C with EQ-5D-3 L and GDS-5 were 0.22 (*p* < 0.01) and 0.45 (*p* < 0.05), respectively. The intraclass correlation coefficient of the SIWB-C over a test-retest interval of two weeks was 0.989.

**Conclusions:**

The SIWB-C was found to be a potential useful measure of subjective spiritual well-being in elderly Taiwanese. Its application in assessing the spiritual well-being in Mandarin-speaking elderly population warrants further investigation.

**Electronic supplementary material:**

The online version of this article (doi:10.1186/s12877-016-0392-1) contains supplementary material, which is available to authorized users.

## Background

In Taiwan, the senior citizens accounted for 11.8% of the total population [1]. It has been estimated that Taiwan will move from an “aging” society to an “aged” one by 2018. The spiritual well-being has become an increasingly important issue for the elderly people.

Gomez and Fisher [2] proposed that spiritual well-being “can be defined in terms of a state of being reflecting positive feelings, behaviors, and cognitions of relationship with oneself, others, the transcendent and nature, that in turn provide the individual with a sense of identity, wholeness, satisfaction, joy, contentment, beauty, love, respect, positive attitudes, inner peace and harmony, and purpose and direction in life” (p. 1976). Spiritual well-being has also been considered as an internal coping resource to buffer the negative effects of uncertainty on psychosocial well-being among individuals with long-term health problems [3]. Doenges and Moorhouse suggested that when one possesses spiritual well-being, he or she can add meaning, purpose, and value to life as well as derive peace, harmony, and contentment [4]. Conversely, low spiritual well-being has been associated with a low quality of life [5] and depression [6] in elderly people.

Given the importance of assessing spiritual well-being, several instruments have been developed for its measurement. In a systematic review, Monod et al. reviewed five instruments designed to assess spiritual well-being in clinical health research setting [7]. The authors concluded that the Functional Assessment of Chronic Illness Therapy – Spiritual Well-Being Scale (FACIT-Sp) [8] and the Spirituality Index of Well-Being (SIWB) [9] were the most well-validated instruments for the assessment of a patient’s current spiritual state. However, a cross-sectional study of 208 elderly patients admitted to a geriatric post-acute rehabilitation unit indicated that the FACIT-Sp might underestimate spiritual well-being in hospitalized elderly patients [10]. On the other hand, the 12-item SIWB was found to be a valid and reliable measure of subjective spiritual well-being in community-dwelling elderly individuals [9]. The SIWB showed significant and expected correlations with other well-validated quality of life measures related to subjective well-being, including the Geriatric Depression Scale (*r* = −0.35), EuroQol (*r* = 0.18), the Physical Functioning Index from the Short Form 36 (*r* = 0.28), and the Years of Healthy Life Scale (*r* = −0.35) [9]. In addition, the conceptualization of SIWB was grounded in a qualitative study of patient perspective of spirituality and well-being [11]. The SIWB was subsequently conceptualized with two independent, but related, dimensions– self-efficacy and life scheme [12]. However, despite being a psychometrically sound measure of spiritual well-being, the utility of SIWB within the Chinese populations is unknown. Therefore, this study translated the SIWB into Chinese and evaluated its psychometric properties among elderly individuals in Taiwan.

## Methods

### Study design and participants

Using a purposive sampling procedure, potential participants were recruited from three different settings: a medical center with 60 geriatric beds, a long-term care institution with 256 elderly residents, and a community health center with approximately 50 elderly visiting each day. They were identified by their treating physicians, health providers, and the community health center. The inclusion criteria included age of 65 years and older, the ability to communicate in Chinese, and had no obvious cognitive impairment as determined by the two research nurses of this study. The study was approved by the Institutional Review Board of the Taichung Veterans General Hospital, Taiwan (No. CE14109). Informed written consent was obtained from all participants.

### Spirituality index of well-being

The SIWB was developed to measure one’s perceptions of their spiritual quality of life. Based on the results of a factor analysis on 277 community-dwelling elderly individuals recruited from primary care clinic sites in the Kansas City metropolitan area, the initial 40-item version of the SIWB was reduced to 12 items, with a Cronbach’s alpha of 0.87 [9]. The SIWB included two domains– 6 items on intrapersonal self-efficacy domain and 6 items on life scheme domain. The self-administered instrument used a 5-point Likert scale ranging from 1 (strongly disagree) to 5 (strongly agree). Higher scores indicated lower levels of spiritual well-being. The original SIWB in English is available as online at http://www.annfammed.org/content/suppl/2004/10/04/2.5.499.DC1/Daaleman_Appendix.pdf.

### Geriatric depression scale-5

The Geriatric Depression Scale-5 (GDS-5) is a useful screening tool with five items for detecting geriatric depression [13]. The five items were: (1) “Are you basically satisfied with your life?”, (2) “Do you often get bored?”, (3) “Do you often feel helpless?”, (4) “Do you prefer to stay at home rather than going out and doing new things?”, and (5) “Do you feel pretty worthless the way you are now?”. A score of 0 to 1 suggests that the respondent is not depressed while 2 or higher is indicative of possible depression. In this study, we used the Chinese version GDS-5 to obtain a depression score for our participants and tested its correlation with our translated Chinese version of the SIWB. In a study of 181 cognitively intact older subjects recruited from a geriatric acute care ward, a geriatric outpatient clinic, and a nursing home in Italy, the GDS-5 had a sensitivity of 0.94, a specificity of 0.81, a positive predictive value of 0.81, and a negative predictive value of 0.94. It also showed a significant agreement with the clinical diagnosis of depression (kappa = 0.74). The interrater reliability (kappa = 0.88) and test-retest reliability (kappa = 0.84) were good [14]. The Chinese version of the GDS-5 had a sensitivity of 0.89, specificity of 0.66, positive predictive value of 0.41, and negative predictive value of 0.95. The Cronbach’s alpha was 0.78 [15].

### Health-related quality of life scale

The EQ-5D-3 L is a measure of self-reported health status developed by the EuroQol Group. It consists of two components: a descriptive classification component and a Visual Analogue Scale (VAS). The descriptive component consists of five dimensions of health, including mobility, self-care, usual activities, pain/discomfort, and anxiety/depression. Each dimension has three levels, namely “no problems”, “some problems”, and “extreme problems” [16, 17]. Perfect health is defined as having no problem in any of the five domains, while the worst possible state is being unable to perform any of the five activities. In this study, we used the descriptive classification component of the Chinese version of the EQ-5D-3 L, which was reported to have a test-retest reliability of 0.51. The Cronbach’s alpha for mobility was 0.98, self-care was 1.0, usual activities was 0.98, pain/discomfort was 0.84, and anxiety/depression was 0.83 [18]. In this study, the concurrent validity of the Chinese version of the SIWB was assessed by the correlations among the scores of EQ-5D-3 L, GDS-5, and Chinese version of the SIWB.

### Translation procedures and psychometric testing

Linguistic validation of the Chinese language SIWB was performed using a standard procedure of translation and blind back-translation. Based on the Brislin’s translation model [19], Phase I involved three steps: (1) forward translation (the translation of SIWB from English into Chinese); (2) back translation (translation of the Chinese version scale back into English); and (3) evaluation of translation equivalence. Permission to translate the original English version of the SIWB to Chinese was obtained from Dr. Timothy P. Daaleman of the original instrument. We utilized the Translation Validity Index (TVI) [20], to obtain systematic judgments from a panel of three experts concerning the translation equivalence between the original English version of the SIWB and the Chinese version of the SIWB. A TVI assessment form that contained a 4-point Likert-type scale (1 = not relevant, 2 = needs major item modification to be equivalent, 3 = equivalent but needs minor modification, and 4 = equivalent) for each item was used. Two scoring criteria were emphasized during Phase I: the translation tried to replicate the original as closely as possible in its meaning and, simultaneously, a sensitive cultural adaptation was employed for items with words that hold different conceptual meaning between cultures [19]. The translated version of the Spiritual Index of Well-Being (SIWB-C) (Additional files 1 and 2) achieved a score of 4 for every item of the entire instrument except one item that received a score of 3. The item was carefully retranslated until its translation equivalence achieved a score of 4.

Phase II was the psychometric testing of the SIWB-C obtained from phase I on Chinese elderly. Participants were recruited from three different settings: a medical center, a long-term care institute, and a community health center in Taiwan.

### Data collection

Two trained research nurses were responsible for administering the questionnaires and collection data from the participants in the three locations: a hospital, a long-term care institution, and a community center. Participants were asked to complete a battery of self-report questionnaires, which consisted of the SIWB-C, the EQ-5D-3 L, the GDS-5 scales, and a questionnaire on socio-demographic information. For participants with low literacy level, each question was read aloud with explanation, if necessary, by the trained research nurses. The verbal responses of the participants were then recorded on the questionnaires. Test-retest reliability was evaluated by administering the SIWB-C to all the participants for a second time one week after the initial administration.

### Statistical analysis

Data were analyzed using IBM SPSS Statistics software package, version 20.0 for Windows (IBM Corp., Armonk, NY, USA). Categorical data were expressed as frequency and percentages. Continuous data were expressed as mean and standard deviation. In addition, face validity of the SIWB-C was tested on nine elderly with an educational level of elementary school. Convergent validity was accessed using Pearson’s correlation coefficients of the SIWB-C, the EQ-5D-3 L, and the GDS-5. The latter two measures were selected for assessing the convergent validity in this study following the original study of the SIWB.

Exploratory factor analysis with Varimax rotation was performed to determine the construct validity. The reliability of the SIWB-C was tested with an internal consistency test based on the Cronbach’s alpha coefficient and a stability test based on the Guttman split-half coefficient. Test-retest reliability was analyzed with intraclass correlation coefficient using a two-way mixed effects model and type consistency.

Confirmatory factor analysis was conducted using AMOS, version 22.0 (SPSS Inc., Chicago, IL, USA) with a maximum likelihood estimate. Average variance extracted (AVE) (values of ≥ 0.50 indicated acceptable) were included for evaluating the convergent validity of the SIWB-C. Structural equation modeling was used to assess the fit 2-factor model compared with the 1-factor model. The goodness-of-fit of the two models were compared using absolute fit index, relative fit index, and parsimony fit index.

## Results

### Description of sample

A total of 416 elderly participants from three different settings were included in this study (149 from a medical center, 150 from a long-term care institution, and 117 from a community health center). Of the 416 participants, 287 (69%) were male and 129 (31%) were female. Their mean age was 81.1 ± 8.4 years. The basic characteristics of the participants are summarized in Table 1.

**Table 1:** Basic characteristics of the study sample (*N* = 416)

Variable	Number	Percent
Setting		
Medical center	149	35.8
Long-term care institution	150	36.1
Community health center	117	28.1
Sex		
Male	287	69.0
Female	129	31.0
Educational level		
Illiterate	48	11.5
Literate	49	11.8
Primary school	176	42.3
Junior high school	43	10.3
Senior high school	45	10.8
University	55	13.2
Marital Status		
Single	71	17.1
Married	204	49.0
Widowed	126	30.3
Separated	5	1.2
Divorced	10	2.4
Religion		
None	181	43.5
Buddhist	97	23.3
Taoist	69	16.6
Christian	39	9.4
Muslin	8	1.9
Catholic	17	4.1
Others	5	1.2
Age (year)		
Mean (standard deviation)	81.1	(8.4)

### Face validity, construct validity, and convergent validity

Face validity, construct validity, and convergent validity were used for the validation of the SIWB-C. To assess face validity, the SIWB-C was given to nine elderly with the educational level of elementary school to understand how they perceived and interpreted the items. The participants reported that the wordings of the SIWB-C were clear and they had little difficulty understanding it.

The construct validity of the SIWB-C was evaluated using exploratory factor analysis. The analysis confirmed the two-dimensional structure of the scale, with factor loadings above 0.30 on all items. The model could explain 68.6% of the total variance. Regarding the convergent validity of SIWB-C, the correlations of SIWB-C with EQ-5D-3 L and GDS-5 were 0.22 (*p* < 0.01) and 0.45 (*p* < 0.05), respectively (Table 2).

**Table 2:** Correlations of the Spiritual Index of Well-Being Chinese version (SIWB-C), EuroQol quality of life scale (EQ-5D-3 L), and Geriatric Depression Scale-5 (GDS-5)

Instrument	SIWB-C	EQ-5D-3 L	GDS-5
SIWB-C	1		
EQ-5D-3 L	0.22**	1	
GDS-5	0.45*	0.45*	1

For SIWB-C, a higher score indicates a lower level of spiritual well-being

For EQ-5D-3 L, a higher score indicates a lower health-related quality of life

For GDS-5, a higher score indicates more symptoms of depression

*SIWB-C* spiritual index of well-being Chinese version, *EQ-5D-3 L* EuroQol quality of life scale, *GDS-5* geriatric depression scale-5

*p < 0.01 **p < 0.01

### Internal consistency reliability, split-half reliability, test-retest reliability, convergent validity, and model fit

The Cronbach’s alpha coefficient for the 12 items of the SIWB-C was 0.94. For the self-efficacy and life scheme subscales, the Cronbach’s alpha coefficients were 0.86 and 0.93, respectively. Table 3 shows the factor loadings, corrected item-total correlations, Cronbach’s alphas if an item was deleted, means, and standard deviations of each item. The Guttman split-half coefficient of SIWB-C was 0.84, indicating that it has adequate reliability. An excellent intra-class correlation coefficient of 0.989 (95% confidence interval = 0.987–0.991) was found over a test-retest interval of two weeks.

**Table 3:** Spiritual Index of Well-Being Chinese version (SIWB-C) items with factor loading, construct reliability, and convergent validity of confirmatory factor analysis (*N* = 416)

Item	Factor loading	Corrected item-total correlation	Cronbach’s *α* if item deleted	Mean	SD	Construct reliability	AVE
Spirituality Index of Well Being Chinese version (Cronbach’s α = 0.934)							
Subscale: self-efficacy (Cronbach’s α = 0.862)						0.82	0.70
1. There is not much I can do to help myself.	0.807	0.427	0.939	2.98	1.12		
2. Often, there is no way I can complete what I have started	0.606	0.660	0.931	3.07	1.22		
3. I can’t begin to understand my problems.	0.447	0.747	0.927	3.20	1.18		
4. I am overwhelmed when I have personal difficulties and problems.	0.617	0.701	0.929	3.20	1.12		
5. I don’t know how to begin to solve my problems.	0.562	0.778	0.926	3.16	1.18		
6. There is not much I can do to make a difference in my life.	0.768	0.582	0.933	2.93	1.14		
Subscale: life scheme (Cronbach’s α = 0.931)						0.83	0.72
7. I haven’t found my life’s purpose yet.	0.756	0.695	0.929	3.10	1.14		
8. I don’t know who I am, where I came from, or where I am going.	0.848	0.744	0.928	3.16	1.28		
9. I have a lack of purpose in my life.	0.836	0.810	0.925	3.15	1.23		
10. In this world, I don’t know my true sense of belonging.	0.838	0.827	0.924	3.08	1.22		
11. I am far from understanding the meaning of life.	0.864	0.780	0.926	3.10	1.24		
12. There is a great void in my life at this time.	0.740	0.763	0.927	3.18	1.27		

*AVE* average variance extracted, *SD* standard deviation

Table 3 also shows an AVE of 0.70 and 0.72 for the self-efficacy and life scheme subscales, respectively, indicating a fine convergent validity of the SIWB-C. Table 4 shows the various indices of goodness-of-fit for the two models. The 2-factor model of SIWB-C showed a more adequate fit than the 1-factor model.

**Table 4:** Model fit indices of the confirmatory factor analysis of the one- and two-factor models

Global model fit index	Acceptable criterion	1-factor model	2-factor model
Absolute fit index			
Likelihood-ratio χ^2^		383.0*	163.73*
Degree of freedom	-	54	53
Goodness of fit index (GFI)	≥ 0.90	0.829	0.935
Adjusted goodness of fit index (AGFI)	≥ 0.90	0.754	0.905
Standardized root mean square residual (SRMR)	≤ 0.08	0.063	0.063 0.041
Root mean square error of approximation (RMSEA)	≤ 0.08	0.121	0.071
Relative fit index			
Normed fit index (NFI)	≥ 0.90	0.890	0.953
No normed fit index (NNFI)	≥ 0.90	0.882	0.960
Relative fit index (RFI)	≥ 0.90	0.866	0.941
Incremental fit index (IFI)	≥ 0.90	0.904	0.968
Comparative fit index (CFI)	≥ 0.90	0.904	0.968
Parsimony fit index			
Parsimony goodness of fit index (PGFI)	≥ 0.50	0.574	0.635
Parsimony normed fit index (PNFI)	≥ 0.50	0.728	0.765
Parsimony comparative fit index (PCFI)	≥ 0.50	0.739	0.777
Likelihood-ratio χ^2^ */*degree of freedom	≤ 3	7.09	3.09

*p < 0.001

## Discussion

The main aim of this study was to translate the English version of the well-validated SIWB to Chinese and examine the psychometric properties of the Chinese version of the SIWB. The participants of this study were recruited from three different settings, namely, a hospital, a long-term care institution, and a community health center. Therefore, the results should be able to represent elderly of a diverse background. Overall, the SIWB-C showed good face validity, construct validity, and internal consistency in our study sample.

Compared with the original English version of the SIBW, the SIWB-C performed well with a Cronbach’s alpha coefficient of 0.94, which is higher than the 0.91 in the English version. Two factors also emerged from the factor analysis with the same items in each factor as the English version. The Cronbach’s alpha coefficients of SIWB-C for the self-efficacy and life scheme subscales were 0.86 and 0.93, indicating high internal consistency similar to the English version. Over 68% of the total variance could be accounted for by the two-factor model and the percentage was higher than the 56% observed in the English version. In addition, the SIWB-C showed significant but moderate levels of convergent validity with EQ-5D-3 L and GDS-5, indicating these measures have unique constructs. Confirmatory factor analysis indicated a fine convergent validity of the SIWB-C and the two-factor model was well fit.

The English version of the SIWB was grounded in a qualitative study of patient perspective of spirituality and well-being. It was not developed based on a religious framework. Spirituality is a universal concept, unique to all people, such as creativity, art, and self expression [21]. The original developer of the SIWB indicated that “the SIWB may be a more culturally sensitive instrument in diverse patient populations because a reference to God is absent” [22]. We concurred with the authors that this point is particularly important for its application in the Chinese population. In Taiwan, faith is characterized by a diversity of beliefs and a high degree of syncretism among folk religion and Chinese traditions. It should also be noted that only 11% of people with a religious belief were actually a formal member of their faith and relatively few people considered themselves a monotheistic [23]. Therefore, the SIWB-C should be more relevant for use in Chinese societies that are often multi-faith in nature, compared with scales that were developed based on formal religious affiliations.

Regarding the translation of the SIWB-C, most items appeared to have culturally equivalent terms in Chinese and we were able to translate without the need for further adaptation. The only exception was the item 10 “In this world, I don’t know where I fit in”. We used the Chinese word for “sense of belonging” instead of “fit in” to capture the cultural connotations.

The SIWB-C should be a valid and reliable instrument for use in Chinese elderly people. Nevertheless, several limitations to the current study should be noted. First, the SIWB-C was evaluated with Mandarin-speaking Taiwanese people. Its psychometric performance in other Chinese populations awaits clarification. Second, 11.5% and 11.8% of the participants were illiterate or with an educational level below primary school, respectively. Although the questions were read aloud and explained by a trained research nurse, it is possible that their responses might be affected by their literacy level. Third, the predictive validity of the SIWB-C was not tested because of the use a cross-sectional study design. Future studies should examine these issues.

## Conclusions

The translated 12-item SIWB-C was found to be a potentially useful measure of subjective spiritual well-being in elderly Taiwanese. Its application in assessing the spiritual well-being in Mandarin-speaking elderly population warrants further investigation.

## Additional files

Additional file 1: Spiritual Index of Well-Being Chinese version (SIWB-C) in Traditional Chinese. (DOC 59 kb)
Additional file 2: Spiritual Index of Well-Being Chinese version (SIWB-C) in Simplified Chinese. (DOC 57 kb)

## Abbreviations

EQ-5D-3 L: EuroQol 5 dimensions 3 levels quality of life scale
FACIT-Sp: Functional assessment of chronic illness therapy – spiritual well-being scale
GDS-5: Geriatric depression scale-5
SIWB: Spirituality index of well-being
SIWB-C: Spirituality index of well-being, Chinese version
TVI: Translation validity index
VAS: Visual analogue scale
