# Supplementary material for: Psychometric properties of the Chinese version of Spiritual Index of Well-Being in elderly Taiwanese
Source: BMC Geriatr. 2017 Jan 4;17:3. doi: 10.1186/s12877-016-0392-1 (PMC5214708; doi:10.1186/s12877-016-0392-1)
Supplement: Additional file 1: — Spiritual Index of Well-Being Chinese version (SIWB-C) in Traditional Chinese. (DOC 59 kb) [file 12877_2016_392_MOESM1_ESM.doc]

**心靈安適量表**

**請選出最符合您的實際情形的選項，並於□中打勾，如 ☑。**

| **項目╱評估** | **非**  **常**  **不**  **同**  **意** | **不**  **同**  **意** | **沒**  **意**  **見** | **同**  **意** | **非**  **常**  **同**  **意** |
| --- | --- | --- | --- | --- | --- |
| **自我效能評估（第1至6項）** |  |  |  |  |  |
| 1. 對於改善自己的現況，我能做的不多。 | □ | □ | □ | □ | □ |
| 2. 我往往將事情進行到一半就無力完成。 | □ | □ | □ | □ | □ |
| 3. 對於自己的問題，我完全沒有頭緒。 | □ | □ | □ | □ | □ |
| 4. 對於我個人的困難或問題，我感到不勝負荷。 | □ | □ | □ | □ | □ |
| 5. 我不知從何開始解決自己的問題。 | □ | □ | □ | □ | □ |
| 6. 對於改變自己的人生，我能做的有限。 | □ | □ | □ | □ | □ |
| **生活規劃評估（第7至12項）** |  |  |  |  |  |
| 7. 我還沒找到人生的目標。 | □ | □ | □ | □ | □ |
| 8. 我不知道自己是誰、來自何處、要往何處去。 | □ | □ | □ | □ | □ |
| 9. 我的人生缺乏目標。 | □ | □ | □ | □ | □ |
| 10.在這世界上，我不知道自己真正歸屬哪裡。 | □ | □ | □ | □ | □ |
| 11.我根本不了解生命的意義。 | □ | □ | □ | □ | □ |
| 12.此刻，我的生命有很大的空虛。 | □ | □ | □ | □ | □ |
