# Supplementary material for: Psychometric properties of the Chinese version of Spiritual Index of Well-Being in elderly Taiwanese
Source: BMC Geriatr. 2017 Jan 4;17:3. doi: 10.1186/s12877-016-0392-1 (PMC5214708; doi:10.1186/s12877-016-0392-1)
Supplement: Additional file 2: — Spiritual Index of Well-Being Chinese version (SIWB-C) in Simplified Chinese. (DOC 57 kb) [file 12877_2016_392_MOESM2_ESM.doc]

**心灵安适量表**

**请选出最符合您的实际情形的选项，并于□中打勾，如☑。**

| **项目╱评估** | **非**  **常**  **不**  **同**  **意**  **(1)** | **不**  **同**  **意**  **(2)** | **没**  **意**  **见**  **(3)** | **同**  **意**  **(4)** | **非**  **常**  **同**  **意**  **(5)** |
| --- | --- | --- | --- | --- | --- |
| **自我效能评估（第1至6项）** |  |  |  |  |  |
| **1. 对于改善自己的现况，我能做的不多。** | □ | □ | □ | □ | □ |
| **2. 我往往将事情进行到一半就无力完成。** | □ | □ | □ | □ | □ |
| **3. 对于自己的问题，我完全没有头绪。** | □ | □ | □ | □ | □ |
| **4. 对于我个人的困难或问题，我感到不胜负荷。** | □ | □ | □ | □ | □ |
| **5. 我不知从何开始解决自己的问题。** | □ | □ | □ | □ | □ |
| **6. 对于改变自己的人生，我能做的有限。** | □ | □ | □ | □ | □ |
| **生活规划评估（第7至12项）** |  |  |  |  |  |
| **7. 我还没找到人生的目标。** | □ | □ | □ | □ | □ |
| **8. 我不知道自己是谁、来自何处、要往何处去。** | □ | □ | □ | □ | □ |
| **9. 我的人生缺乏目标。** | □ | □ | □ | □ | □ |
| **10.在这世界上，我不知道自己真正归属哪里。** | □ | □ | □ | □ | □ |
| **11.我根本不了解生命的意义。** | □ | □ | □ | □ | □ |
| **12.此刻，我的生命有很大的空虚。** | □ | □ | □ | □ | □ |
